# Performance of a HER2 testing algorithm specific for p53‐abnormal endometrial cancer

**DOI:** 10.1111/his.14381

**Published:** 2021-07-05

**Authors:** Lisa Vermij, Naveena Singh, Alicia Leon‐Castillo, Nanda Horeweg, Jan Oosting, Joseph Carlson, Vincent Smit, Blake Gilks, Tjalling Bosse

**Affiliations:** ^1^ Department of Pathology Leiden University Medical Center Leiden the Netherlands; ^2^ Department of Pathology Barts Health NHS Trust London UK; ^3^ Department of Radiation Oncology Leiden University Medical Center Leiden the Netherlands; ^4^ Department of Oncology‐Pathology Karolinska Institutet Stockholm Sweden; ^5^ Department of Pathology and Laboratory Medicine University of British Columbia Vancouver Canada

**Keywords:** endometrial cancer, HER2, immunohistochemistry, *in‐situ* hybridisation, interobserver variability

## Abstract

**Aims:**

Human epidermal growth factor receptor 2 (HER2) amplification in endometrial cancer (EC) is almost completely confined to the p53‐abnormal (p53abn) molecular subtype and independent of histological subtype. HER2 testing should therefore be molecular subtype‐directed. However, the most optimal approach for HER2 testing in EC has not been fully established. Therefore, we developed an EC‐specific HER2 immunohistochemistry (IHC) scoring method and evaluated its reproducibility and performance to establish an optimal diagnostic HER2 testing algorithm for p53abn EC.

**Methods and results:**

HER2 IHC slides of 78 p53abn EC were scored by six gynaecopathologists according to predefined EC‐specific IHC scoring criteria. Interobserver agreement was calculated using Fleiss’ kappa and the first‐order agreement coefficient (AC1). The consensus IHC score was compared with HER2 dual *in‐situ* hybridisation (DISH) results. Sensitivity and specificity were calculated. A substantial interobserver agreement was found using three‐ or two‐tiered scoring [κ = 0.675, 95% confidence interval (CI) = 0.633–0.717; AC1 = 0.723, 95% CI = 0.643–0.804 and κ = 0.771, 95% CI = 0.714–0.828; AC1 = 0.774, 95% CI = 0.684–0.865, respectively]. Sensitivity and specificity for the identification of HER2‐positive EC was 100 and 97%, respectively, using a HER2 testing algorithm that recommends DISH in all cases with moderate membranous staining in >10% of the tumour (IHC+). Performing DISH on all IHC‐2+ and ‐3+ cases yields a sensitivity and specificity of 100%.

**Conclusions:**

Our EC‐specific HER2 IHC scoring method is reproducible. A screening strategy based on IHC scoring on all cases with subsequent DISH testing on IHC‐2+/‐3+ cases has perfect test accuracy for identifying HER2‐positive EC.

## Graphical abstract

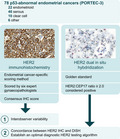

## Introduction

Human epidermal growth receptor 2 (HER2) has gained interest as a biomarker in endometrial cancer (EC) with the potential to predict response to (adjuvant) anti‐HER2 therapies. A recent Phase II clinical trial including advanced and recurrent HER2‐positive serous EC showed significantly improved progression‐free and overall survival for patients receiving combined treatment of chemotherapy and trastuzumab.^1^, ^2^ These promising results merit further exploration of anti‐HER2 therapies in EC. Hence, it will become increasingly important to have a robust HER2 testing algorithm that can be applied in EC to select patients.

In general, HER2‐positive tumours can be identified by HER2 immunohistochemistry (IHC) with or without subsequent *in‐situ* hybridisation (ISH) on equivocal cases. For breast and gastric cancer, tumour‐specific HER2 testing guidelines have been developed.^3^, ^4^ Most studies investigating HER2‐status in EC have used the Food and Drug Authority (FDA) criteria for HER2 testing in breast cancer.^5^ It is well described that HER2‐positive EC, more frequently than HER2‐positive breast cancer, shows incomplete membranous staining and intratumoral HER2 heterogeneity.^6^, ^7^ A significant proportion of HER2‐positive EC will be misclassified if the breast cancer‐specific HER2 testing guidelines are applied, because incomplete membranous staining in breast cancer is classified as IHC‐1+ and considered to be HER2‐negative. Recently, serous EC‐specific HER2 testing criteria were proposed based on the inclusion criteria of the above‐mentioned Phase II clinical trial.^8^ Cases with strong membranous staining in <30% of the tumour cells or moderate membranous staining in >10% of the tumour cells, regardless of completeness, are considered equivocal (IHC‐2+). Cases with strong membranous staining in >30% of the tumour are considered to be HER2‐positive (IHC‐3+). DISH should be performed to establish the definite HER2 status in the IHC‐2+ category.

Recent work showed that HER2‐positivity can be present in all histological subtypes, but is almost exclusively limited to p53‐abnormal (p53abn) EC.^9^ In the PORTEC‐3 (Adjuvant Chemoradiotherapy Versus Radiotherapy Alone in Women With High‐Risk Endometrial Cancer) trial, all but one HER2‐positive cases were p53abn EC, including a diversity of histologic subtypes (serous*, n* = 9, 37.5%; endometrioid, *n* = 6, 25.0%; clear cell, *n* = 5, 20.8%).^9^ In The Cancer Genome Atlas (TCGA) cohort, HER2 gene amplification was exclusively confined to the copy number (CN)‐high molecular subgroup.^10^, ^11^ Finally, a study including 238 non‐hypermutant *TP53*‐mutant EC recently found that *ERBB2* amplification was present in 17.2% of the cases. No difference was observed in the frequency of *ERBB2* amplification between different histological subtypes.^12^ These findings support the use of the molecular EC classification to direct HER2 testing in EC as opposed to histological subtype‐directed HER2 testing. It is probably the most efficient approach to capture most, if not all, HER2‐positive EC. Nevertheless, future clinical trials should evaluate the benefit of anti‐HER2 therapies in all HER2‐positive p53abn EC.

We propose, based on the present literature, a simplified EC‐specific HER2 IHC scoring method that relies upon membranous staining intensity independent of the completeness of membranous staining.^6^, ^7^, ^8^ First, we describe the interobserver agreement of this EC‐specific HER2 IHC scoring method in a large cohort of p53abn EC. Secondly, we use the consensus HER2 IHC scores to assess concordance with HER2 dual *in‐situ* hybridisation (DISH) to generate an optimal diagnostic HER2 testing algorithm.

## Methods

### Patient and Tissue Selection

**Figure 1:**
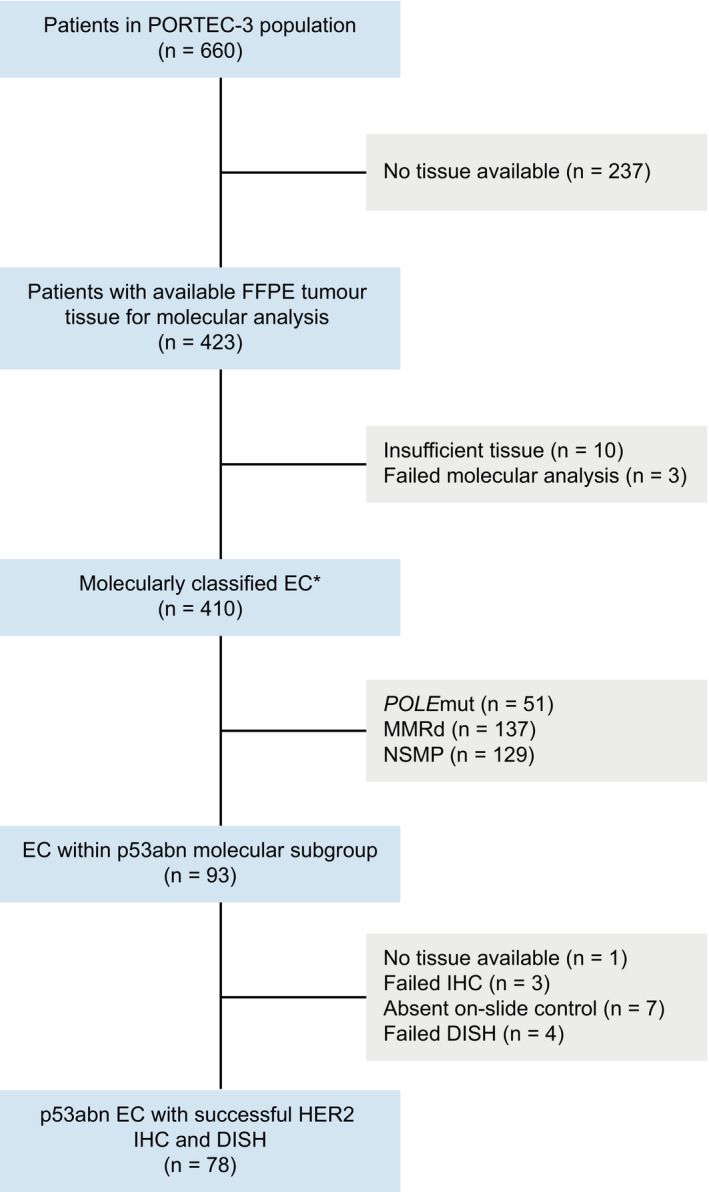
Flowchart of patient selection. *Classified according to the diagnostic algorithm for molecular endometrial cancer classification.^29^ FFPE, formalin‐fixed paraffin‐embedded; EC, endometrial cancer; *POLE*mut, *POLE*‐(ultra)mutated; MMRd, Mismatch repair‐deficient; NSMP, No specific molecular profile; p53abn, p53‐abnormal; IHC, Immunohistochemistry; DISH, Dual *in‐situ* hybridisation.

The cohort comprised 78 p53abn high‐risk EC derived from the PORTEC‐3 clinical trial collected by the *Trans*PORTEC group (Figure 1). Detailed information on the PORTEC‐3 trial design and results have been reported previously.^13^, ^14^ Briefly, the PORTEC‐3 trial recruited 660 patients with International Federation of Gynaecology and Obstetrics (FIGO) 2009 stage IA grade 3 endometrioid EC (EEC) with documented lymphovascular space invasion (LVSI); stage IB grade 3 EEC; stages II–III EEC; or non‐endometrioid EC with stages IA (with invasion), IB, II or III. Upfront pathology review was performed by reference gynaecopathologists to confirm eligibility. Molecular classification [including *POLE*, mismatch repair (MMR) and p53 testing] was successful for 410 patients.^15^ The study was approved by the Dutch Cancer Society and medical ethics committees at participating centres. Written informed consent was obtained from all patients.

### Immunohistochemical Staining of HER2

HER2 IHC staining was performed on 4‐μm slides with the Ventana BenchMark GX (Roche Diagnostics, Basel, Switzerland) using the anti‐HER2/*neu* (4B5) rabbit monoclonal primary antibody and the Ventana *ultra*View dianinobenzidine (DAB) detection kit. Detailed information on the staining protocol has been described previously.^9^ A positive external control was mounted on each individual slide.

### Evaluation of HER2 Immunohistochemistry

For the purpose of this study we used the serous EC‐specific HER2 IHC scoring approach by Buza *et al*.,^8^ with the adjustment that we used intensity of membranous staining to differentiate between 2+ (moderate) and 3+ (strong), and unifying the threshold for both to 10%. All cases with staining in <10% we categorised as IHC‐0. Representative examples of absent, faint, moderate and strong membranous HER2 staining intensities in EC were provided (see Figure S1). Six expert gynaecopathologists (N.S., A.L.C., J.C., V.S., B.G. and T.B.) scored each HER2‐stained slide blinded to the HER2 DISH results. All HER2 IHC slides were scanned at ×40 magnification using the Pannoramic 250 Flash III scanner (3DHistech, Budapest, Hungary) and uploaded onto a website specifically designed for this study. Prior to the study, all observers were simultaneously instructed on the use of the website and informed about the predefined HER2 IHC scoring criteria (Table 1). After all observers completed the survey, a consensus meeting was held. For cases with discrepancies between IHC‐0 and IHC‐1+ scores, the consensus score was based on the majority vote. All other discordant cases were discussed during the consensus meeting after which a final HER2 IHC consensus score was determined.

**Table 1:** Endometrial cancer‐specific HER2 immunohistochemistry scoring criteria

Category	Definition
IHC‐0	No reactivity or membranous reactivity in <10% of tumour cells
IHC‐1+	Faint membranous reactivity in ≥10% of tumour cells
IHC‐2+	Moderate membranous reactivity in ≥10% of tumour cells
IHC‐3+	Strong membranous reactivity in ≥10% of tumour cells

IHC, Immunohistochemistry; HER2, Human epidermal growth factor receptor 2.

### HER2 Dual *In‐Situ* Hybridisation

To determine *HER2* amplification status, HER2 DISH was performed on all cases using the INFORM HER2 dual ISH DNA probe cocktail assay on the Ventana BenchMark GX (Roche Diagnostics). Detailed information of the staining procedure has been described previously.^9^ For each slide, HER2 probe and chromosome enumerating probe (CEP17) signals were counted in at least 20 nuclei and the HER2:CEP17 ratio was calculated. HER2 amplification was defined as a HER2:CEP17 ratio ≥2.0, present in at least 10% of the complete tumour. The HER2 IHC slides were used to direct DISH scoring in the area(s) with strongest membranous staining. Additional areas of the tumour were only screened for the presence of HER2 amplification. The HER2 DISH slides were scored by two observers, other than the observers participating in the interobserver study. Discordant DISH scores between both observers were re‐evaluated until consensus was reached.

### Statistical Analysis

Statistical analyses were performed with spss (Statistical Package of Social Science) version 25 and R version 3.6.1 (http://www.r‐project.org/) using the irrCAC package. Associations between groups were analysed using Fisher’s exact test for categorical variables and the Mann–Whitney *U*‐test for continuous variables. The extent of interobserver agreement was analysed using Fleiss’ kappa and the first‐order agreement coefficient (AC1).^16^, ^17^ The resulting kappa and AC1 values were interpreted accordingly: 0.01–0.20 slight agreement; 0.21–0.40 fair agreement; 0.41–0.60 moderate agreement; 0.61–0.80 substantial agreement; 0.81–1.00 almost perfect agreement.^18^ The performance of the proposed EC‐specific IHC scoring method was determined by calculating the sensitivity, specificity and accuracy of HER2 IHC compared to HER2 amplification status by DISH. A two‐sided *P* < 0.05 was considered statistically significant.

## Results

Clinicopathological characteristics of the 78 p53abn EC and their relationship with HER2 status by DISH are provided in Table 2. Nineteen cases (24.4%) were HER2‐positive by DISH. Age, histotype, grade and specimen type did not differ significantly between patients with HER2‐positive and HER2‐negative p53abn HREC. HER2 testing was performed on surgical resection specimens in 72 patients (92.3%) and on endometrial curettage/biopsy specimens in six patients (7.7%).

**Table 2:** Clinicopathological characteristics of patients with p53‐abnormal endometrial cancer in the PORTEC‐3 trial

Characteristic	Total	DISH not amplified	DISH amplified	*P*‐value
	*n* = 78 (100%)	*n* = 59 (75.6%)	*n* = 19 (24.4%)	
Age, years				
Mean (range)	66.1 (47.3–80.5)	65.2 (47.3–77.0)	68.6 (55.8–80.5)	0.08
Histotype within p53abn EC				
Low grade endometrioid	4 (5.1)	3 (5.1)	1 (5.3)	0.47
High grade endometrioid	18 (23.1)	13 (22.0)	5 (26.3)	
Serous	40 (51.3)	32 (54.2)	8 (42.1)	
Clear cell	10 (12.8)	7 (11.9)	3 (15.8)	
Mixed EEC‐SEC	3 (3.8)	3 (3.8)	0 (0.0)	
Mixed EEC‐CCC	2 (2.6)	1 (1.7)	1 (5.3)	
Other	1 (1.3)	0 (0.0)	1 (5.3)	
Stage				
IA	18 (23.1)	13 (22.0)	5 (26.3)	0.63
IB	12 (15.4)	9 (15.3)	3 (15.8)	
II	20 (25.6)	15 (25.4)	5 (31.6)	
III	28 (35.9)	22 (37.3)	6 (31.6)	
Specimen type				
Curettage/biopsy	6 (7.7)	4 (6.8)	2 (10.5)	0.63
Resection	72 (92.3)	55 (93.2)	17 (89.5)	

DISH, Dual *in‐situ* hybridisation; p53abn, p53‐abnormal; EC, Endometrial cancer; EEC, Endometrioid endometrial cancer; SEC, Serous endometrial cancer; CCC, Clear‐cell carcinoma; PORTEC‐3, Adjuvant Chemoradiotherapy Versus Radiotherapy Alone in Women With High‐Risk Endometrial Cancer.

### Interobserver Agreement of HER2 IHC Scoring

**Figure 2:**
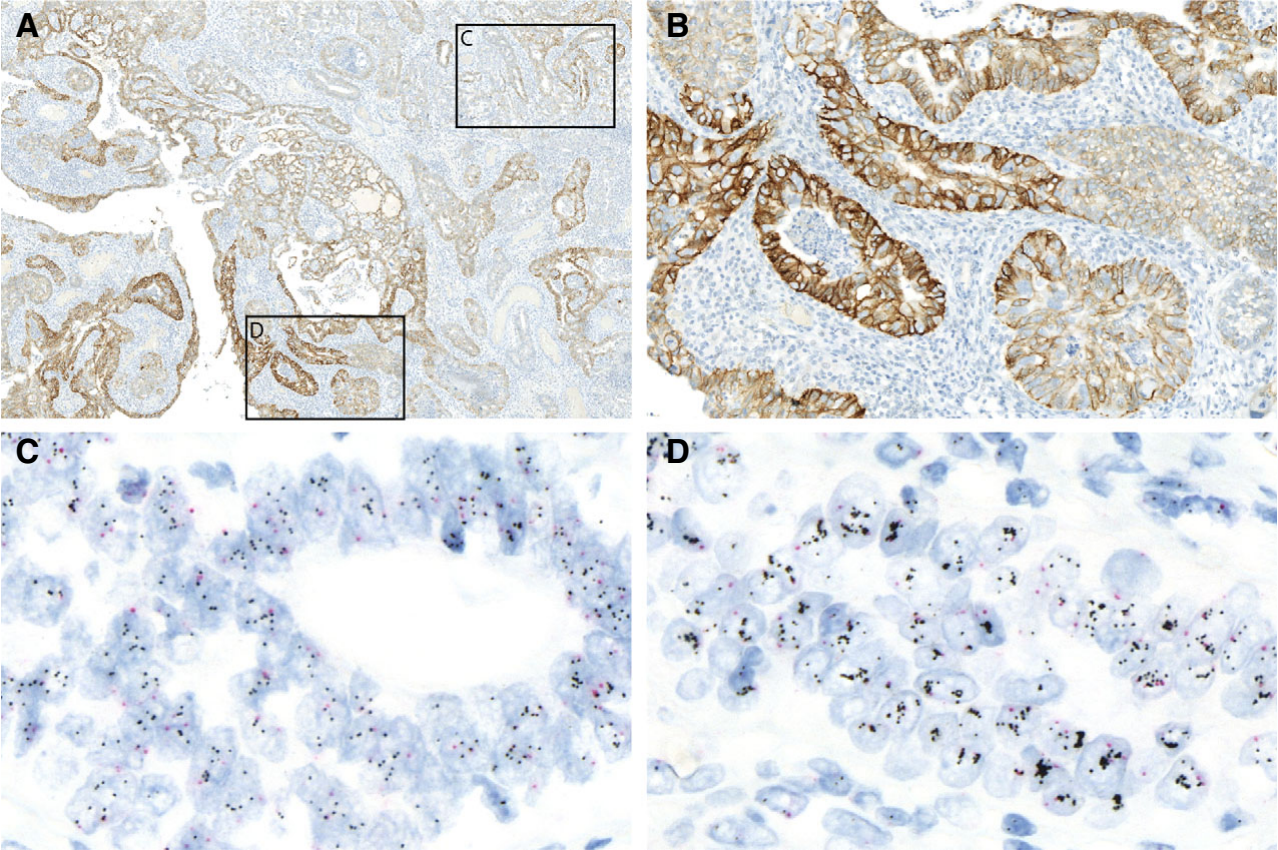
p53‐abnormal endometrial cancer (EC) with heterogeneous human epidermal growth factor receptor 2 (HER2) immunohistochemistry (IHC) staining intensity, with consensus score 2+ and diffuse amplification by dual *in‐situ* hybridisation (DISH). **A**,**B**, HER2 IHC stain of case 38 scored as IHC‐2+ by five observers and IHC‐3+ by one observer (consensus IHC score 2+). There is a large tumour area with moderate intensity (**A**) and a distinct area (<10%) with strong staining (**B**). **C**,**D**, DISH showed diffuse amplification in both areas with moderate staining [**C**; HER2: chromosome enumerating probe (CEP17) ratio = 2.8] and area with strong staining (**D**; HER2:CEP17 ratio = 5.4).

There was complete agreement in 32 cases (41.0%). These cases were scored as followed: IHC‐0 *n* = 20, IHC‐2+ *n* = 5, IHC‐3+ *n* = 7 (see Table S1). Twelve cases had discrepant scores between IHC‐0 and ‐1+ and the final consensus score for these cases was determined by the majority vote (IHC‐0 *n* = 7, IHC‐1+ *n* = 5). The remaining 34 discrepant cases were discussed at the consensus meeting. The discordances encountered could broadly be divided into three categories. First, there were cases that displayed borderline IHC‐2+/‐3+ in terms of intensity; secondly, cases in which the extent of tumour cell staining was around the 10% threshold in cases with heterogeneous staining intensity throughout the tumour (Figure 2); and thirdly, cases that showed staining artefacts such as strong cytoplasmic or nuclear HER2 staining (see Figure S2). The final consensus IHC scores were distributed as follows: IHC‐0 *n* = 30 (38.5%), IHC‐1+ *n* = 13 (16.7%), IHC‐2+ *n* = 21 (26.9%) and IHC‐3 *n* = 14 (17.9%) (Table 3). Individual scores of observers for all cases are provided in the Figure S1. Finally, we found a moderate interobserver agreement using four IHC scoring categories (IHC‐0 versus ‐1+ versus ‐2+ versus ‐3+), and substantial interobserver agreement using three or two HER2 IHC scoring categories (Table 4).

**Table 3:** Comparison of HER2 immunohistochemistry and HER2 dual *in‐situ* hybridisation results

HER2 IHC consensus score	HER2 DISH		
	Not amplified (*n*)	Amplified (*n*)	Total (*n*)
IHC‐0	30	0	30
IHC‐1+	13	0	13
IHC‐2+	14	7	21
IHC‐3+	2	12	14
Total	59	19	78

DISH, Dual *in‐situ* hybridisation; IHC, Immunohistochemistry; HER2, Human epidermal growth factor receptor.

**Table 4:** Interobserver agreement of endometrial cancer‐specific HER2 IHC scoring criteria in p53‐abnormal endometrial cancer

	Fleiss’ kappa	AC1
4 IHC categories		
0, 1+, 2+, 3+	0.565 (95% CI = 0.530–0.600)	0.593 (95% CI = 0.507–0.679)
3 IHC categories		
0/1+, 2+, 3+	0.675 (95% CI = 0.633–0.717)	0.723 (95% CI = 0.643–0.804)
2 IHC categories		
0/1+, 2/3+	0.771 (95% CI = 0.714–0.828)	0.774 (95% CI = 0.684–0.865)

AC1, First‐order agreement coefficient; IHC, Immunohistochemistry; HER2, Human epidermal growth factor receptor; CI, Confidence interval.

### Agreement of HER2 IHC With DISH

**Figure 3:**
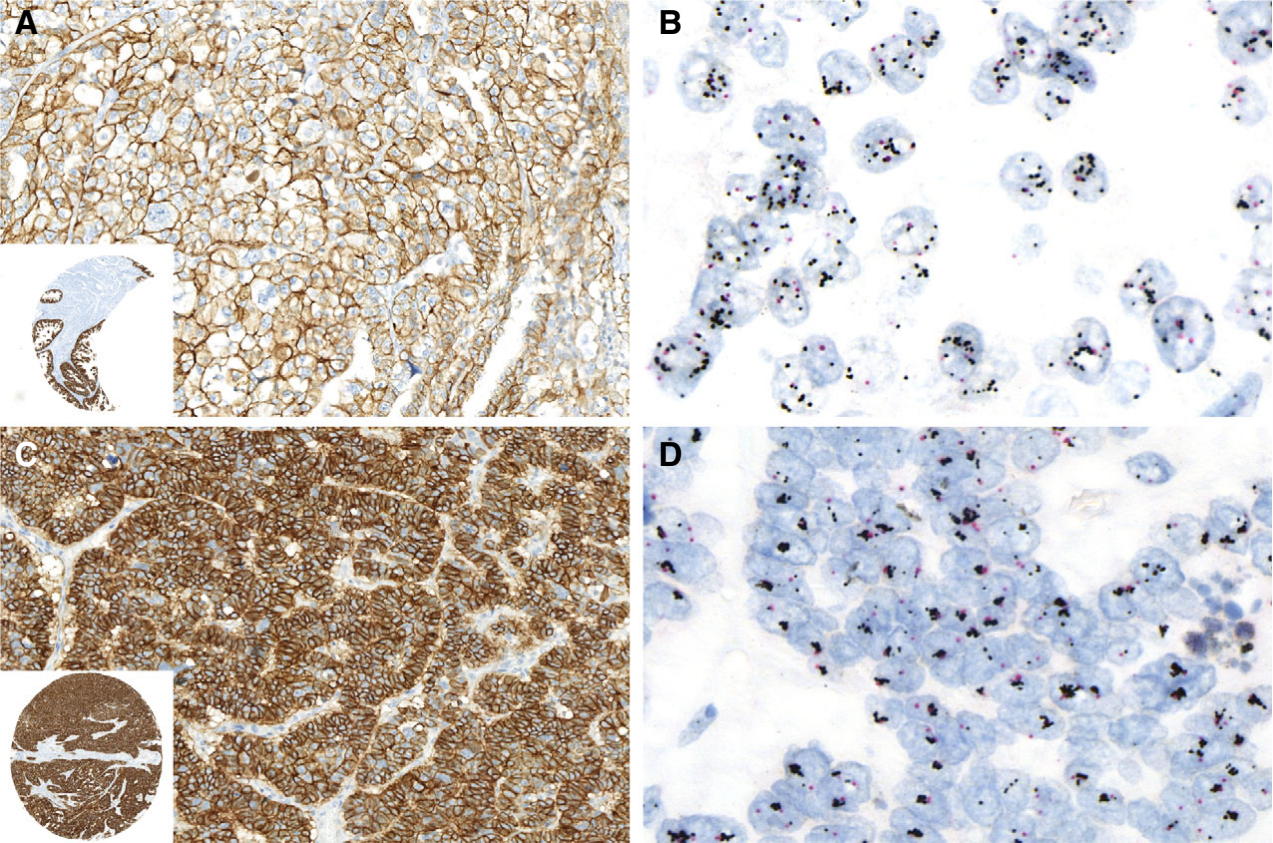
Human epidermal growth factor receptor 2 (HER2) immunoreactivity score may vary in dual *in‐situ* hybridisation (DISH) amplified p53abn endometrial cancer (EC). **A**, HER2 immunohistochemistry (IHC) stain of case 44 scored as IHC‐2+ (by consensus). Inset shows the on‐slide control. **B**, DISH showing HER2 amplification [HER2:chromosome enumerating probe (CEP17) ratio = 4.2]. **C**, HER2 IHC stain of case 58 scored as IHC‐3+ (by consensus). **D**, DISH showing HER2 amplification (HER2:CEP17 ratio = 5.5).

**Figure 4:**
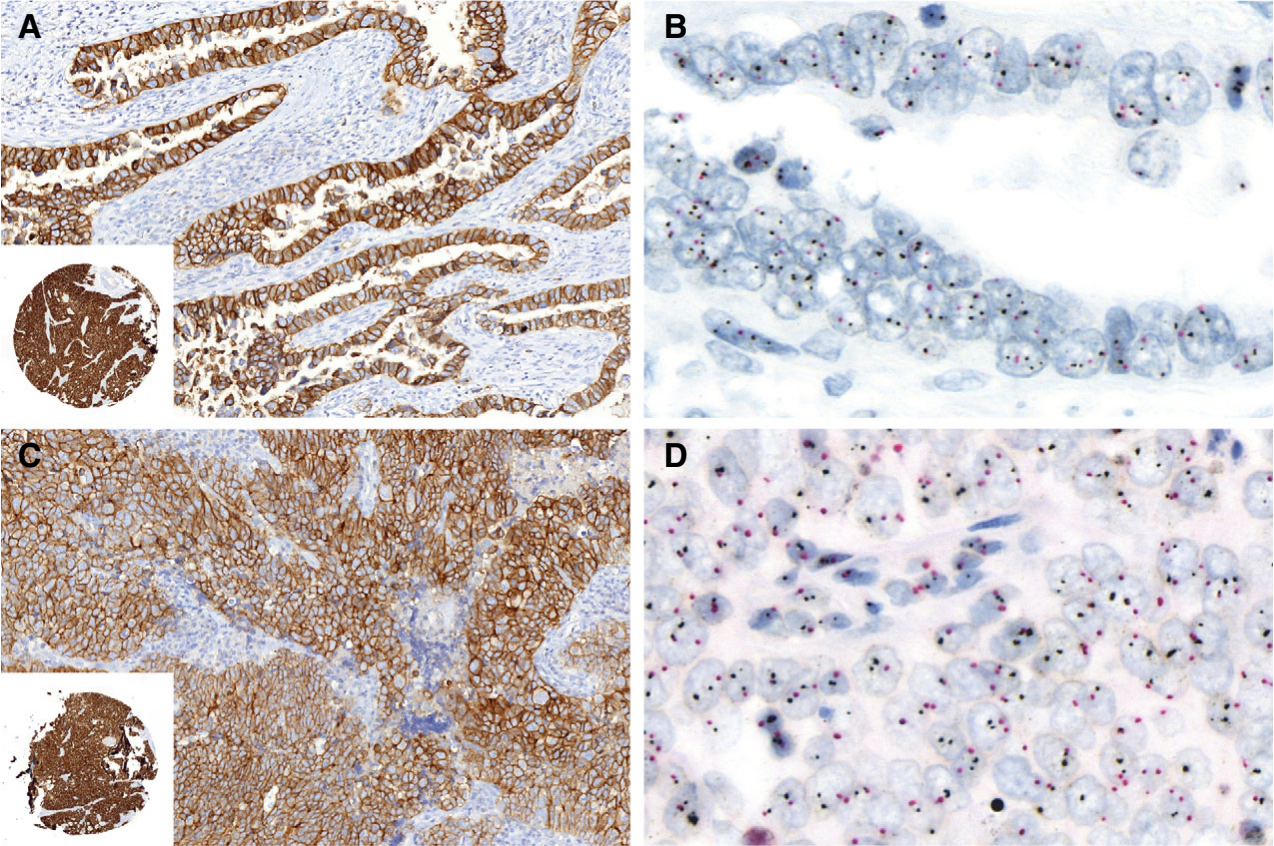
Two p53‐abnormal endometrial cancers (EC) with consensus human epidermal growth factor receptor 2 (HER2) immunohistochemistry (IHC)‐3+ score without amplification by dual *in‐situ* hybridisation (DISH). **A**, HER2 IHC stain of case 1. Inset shows the on‐slide control. **B**, DISH showing no amplification [HER2:chromosome enumerating probe (CEP17) ratio = 1.2]. **C**, HER2 IHC stain of case 74. Inset shows the on‐slide control. **D**, DISH showing no amplification (HER2:CEP17 ratio = 1.1).

The HER2 IHC consensus scores were used to evaluate the agreement with HER2 amplification status by DISH (Table 3). All cases scored as IHC‐0 or ‐1+ did not have HER2 amplification. Examples of DISH‐amplified EC that scored IHC‐2+ and ‐3+ are shown in Figure 3. Considering an IHC‐3+ score as HER2‐positive, HER2 IHC showed an accuracy of 88% [95% confidence interval (CI) = 81–96%] (sensitivity: 63%, 95% CI = 53–74% and specificity: 97%, 95% CI = 93–100%). Importantly, two cases (16 and 73) were scored IHC‐3+ by consensus but did not show HER2 amplification by DISH (Figure 4). In both these cases the tumours showed 100% moderate to strong membranous staining intensity, and the discussion during the consensus meeting regarded whether the intensity was sufficient to allow an IHC‐3+ score. In retrospect, despite the consensus for an IHC‐3+ score, the staining intensity of the on‐slide control appears somewhat stronger than the intensity of the tumour and thus favours an IHC‐2+ score. Two cases (44 and 76) showed strong membranous staining in fewer than 10% of tumour cells and were thus scored IHC‐0. Both cases showed HER2 amplification by DISH in the same area as the strong IHC staining, comprising far less than 10% of the tumour.

### HER2 Testing Algorithm

To determine the optimal strategy for the combined use of HER2 IHC and DISH for detection of HER2‐positive EC, we compared the performance of two different HER2 testing algorithms: (1) HER2 IHC performed on all cases and subsequent DISH testing on cases with IHC‐2+ and ‐3+ scores and (2) HER2 IHC performed on all cases and subsequent DISH testing on cases with IHC‐2+ scores (Table 5). In both scenarios DISH was used as the reference test. In the first scenario, the HER2 testing algorithm had a 100% accuracy, sensitivity and specificity. However, 14 DISH tests (17.9%) would be required to identify two (2.6%) IHC‐3+ cases without HER2 amplification. In the second scenario, the HER2 testing algorithm had an accuracy of 97% (95% CI = 94–100%), a sensitivity of 100% and a specificity of 97% (95% CI = 93–100%). In this scenario, 40% less DISH testing would be required (IHC‐2+ *n* = 21 versus IHC‐2+/‐3+ *n* = 35) at the cost of two cases (14.3%), which would erroneously be classified as HER2‐positive.

**Table 5:** Performance of two endometrial cancer‐specific HER2 testing algorithms

	HER2 IHC on all cases with DISH performed on cases scored	
	IHC‐2+/‐3+	IHC‐2+
Sensitivity	100%	100%
Specificity	100%	97%
Accuracy	100%	97%
Positive predictive value	100%	90%
Negative predictive value	100%	100%

IHC, Immunohistochemistry; DISH, Dual *in‐situ* hybridisation; HER2, Human epidermal growth factor receptor.

## Discussion

Our study shows that our EC‐specific HER2 IHC scoring method has a substantial interobserver agreement among gynaecopathologists using three‐ or two‐tiered scoring (κ = 0.675, 95% CI = 0.633–0.717; AC1 = 0.723, 95% CI = 0.643–0.804 and κ = 0.771, 95% CI = 0.714–0.828; AC1 = 0.774, 95% CI = 0.684–0.865, respectively). In combination with DISH testing on all cases with an IHC‐2+ and/or ‐3+ score, this HER2 IHC scoring method is highly sensitive in the detection of HER2‐amplified p53abn EC. We have made the scoring website used in this study publicly available for self‐assessment to enhance recognition and interpretation of HER2 IHC staining patterns in EC (https://her2.leidenpastudy.nl/).

Despite the lack of specific experience in HER2 scoring in EC, our six gynaecopathologists showed that the EC‐specific IHC soring method used in this study is well reproducible. Interobserver agreement of HER2 scoring in breast cancer is reported to have kappa‐values between 0.49 and 0.80 using a four‐tiered scoring method.^19^, ^20^, ^21^, ^22^, ^23^ The interobserver agreement of HER2 scoring in gastro‐oesophageal cancers is comparable to our findings, with reported kappa‐values between 0.61 and 0.78.^24^, ^25^, ^26^ Thus, the EC‐specific HER2 IHC scoring method that we used, adapted and simplified from the method that was recently proposed for serous EC^8^ is well reproducible among gynaecopathologists, and the interobserver agreement is comparable to that of breast and gastric cancer. Finally, during the final preparation of this manuscript, a similar study reported comparable interobserver agreement using a HER2 testing algorithm adapted from the clinical trial by Fader *et al*.^27^ The study was limited to a small cohort of serous EC and their findings may therefore not be directly applicable to all EC. Furthermore, fluorescence *in‐situ* hybridisation (FISH) was performed only on a subset of cases, hampering evaluation of the concordance between HER2 IHC and ISH in EC.

The relevant tumour percentage cut‐off for final HER2 status determination in EC remains to be clinically defined. Buza *et al*. recently proposed a 30% cut‐off for an IHC‐3+ score, as this threshold was used in the Phase II trial by Fader *et al*. among HER2‐positive advanced and recurrent serous EC patients. In addition, Buza *et al*. found a higher concordance between HER2 IHC and FISH results using the American Society of Clinical Oncology/College of American Pathologists (ASCO/CAP) 2007 HER2 breast cancer guideline (which uses a 30% cut‐off) compared to the original FDA criteria (using a 10% cut‐off) in a study including 52 serous EC.^7^ Given the high frequency of intratumor HER2 heterogeneity in EC it is possible that FISH was not scored in the same area(s) of the tumour that shows IHC‐3+ staining, resulting in discordant IHC and FISH results. In this study, we used the 10% staining threshold following the ASCO/CAP 2018 HER2 breast cancer guideline. Our choice to use 10% in this study is twofold. First, subclonal HER2 overexpression is frequently observed in p53abn EC and represents true HER2 amplification.^9^ Secondly, a significant proportion of p53abn EC show moderate to strong membranous HER2 immunostaining within the range of 10–30% tumour staining. Including these cases in future targeted trials will help to define the true lower threshold of clinically relevant HER2 overexpression.

**Figure 5:**
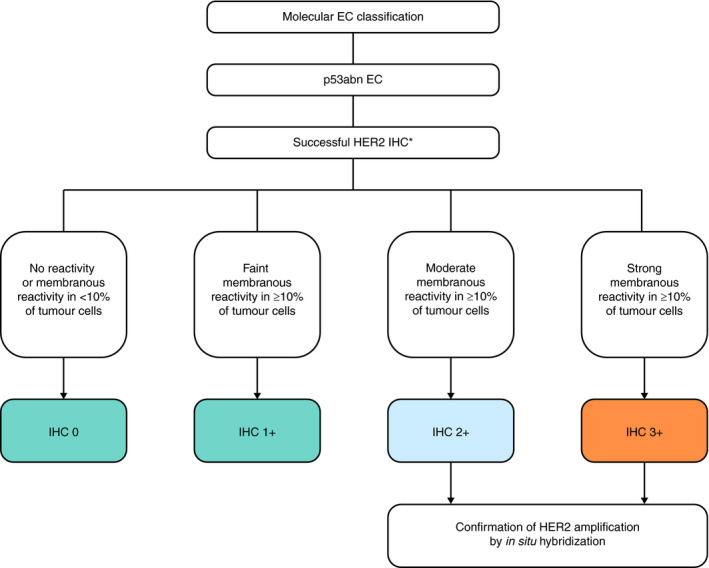
Proposed endometrial cancer‐specific human epidermal growth factor receptor 2 (HER2) testing algorithm for HER2 status assignment in p53‐abnormal endometrial cancer. *Successful immunohistochemistry with on‐slide control showing appropriate staining. EC, Endometrial cancer; p53abn, p53‐abnormal; IHC, Immunohistochemistry.

We have investigated the performance of two different HER2 testing strategies in detecting HER2‐amplified p53abn EC. In the first strategy, HER2 IHC is performed in all cases with subsequent DISH testing on cases scored IHC‐2+ and ‐3+ (depicted in Figure 5). The second strategy suggests subsequent DISH testing only on cases scored IHC‐2+. Using the first strategy, two cases would be identified with discordant results between HER2 IHC and DISH. These cases were scored IHC‐3+ by consensus; however, no HER2 amplification was observed by DISH, resulting in conflicting interpretation of the HER2 status and subsequent treatment recommendations. In breast cancer it is known that HER2 IHC and ISH are equally predictive of response to trastuzumab; however, IHC is superior in predicting treatment response in gastroesophageal cancer.^3^, ^28^ Currently, the only endometrial cancer‐specific HER2 scoring criteria with proven correlation with clinical response is based on the clinical trial.^1^, ^2^ Here, patients with serous EC scored as IHC‐3+ were eligible without confirmation of HER2 amplification status by DISH. Future prospective clinical trials will need to validate the predictive capacity of both IHC and FISH for anti‐HER2 treatment response in HER2‐positive EC patients.

In conclusion, this study demonstrates that a simplified, intensity‐based EC‐specific HER2 IHC scoring method is well reproducible among gynaecopathologists. In addition, we report two highly sensitive testing algorithms for identifying HER2‐positive EC using subsequent DISH testing on either only IHC‐2+ cases or all IHC‐2+ and ‐3+ cases. The clinical utility of these proposed HER2 testing algorithms will need to be validated in a prospective clinical trial on anti‐HER2 treatments in EC patients.

## Conflict of interest

The authors declare no conflicts of interest.

## Supporting information


**Figure S1**. Representative examples of absent, faint, moderate and strong membranous HER2 immunoreactivity in endometrial cancer. Representative examples of HER2 immunohistochemistry with (**A**) absent membranous immunoreactivity, (**B**) faint membranous immunoreactivity, (**C**) moderate membranous immunoreactivity, and (**D**) strong membranous immunoreactivity.

**Figure S2**. Two examples of potential pitfalls in HER2 IHC scoring in p53abn EC. (**A**) HER2 IHC of case #28 scored as IHC 1+ (by consensus), showing weak/moderate immunoreactivity in the basal membrane only (×40). (**B**) No amplification by DISH (HER2:CEP 17 ratio = 1.1; ×120). (**C**) HER2 IHC of case #41 scored as IHC 0 (by consensus), showing strong nuclear and cytoplasmic staining (×40). (**D**) No amplification by DISH (HER2:CEP 17 ratio = 1.1; ×90).

**Table S1**. Detailed description of individual HER2 immunohistochemistry scores per observer, consensus IHC score and dual in situ hybridization results per case.

## Data Availability

The data presented in this study are available upon request from the corresponding author.
