# Supplementary material for: Performance of a HER2 testing algorithm specific for p53‐abnormal endometrial cancer
Source: Histopathology. 2021 Jul 5;79(4):533–43. doi: 10.1111/his.14381 (PMC8518500; doi:10.1111/his.14381)

Figure S1. Representative examples of absent, faint, moderate and strong membranous HER2 immunoreactivity in endometrial cancer. Representative examples of HER2 immunohistochemistry with (A) absent membranous immunoreactivity, (B) faint membranous immunoreactivity, (C) moderate membranous immunoreactivity, and (D) strong membranous immunoreactivity.

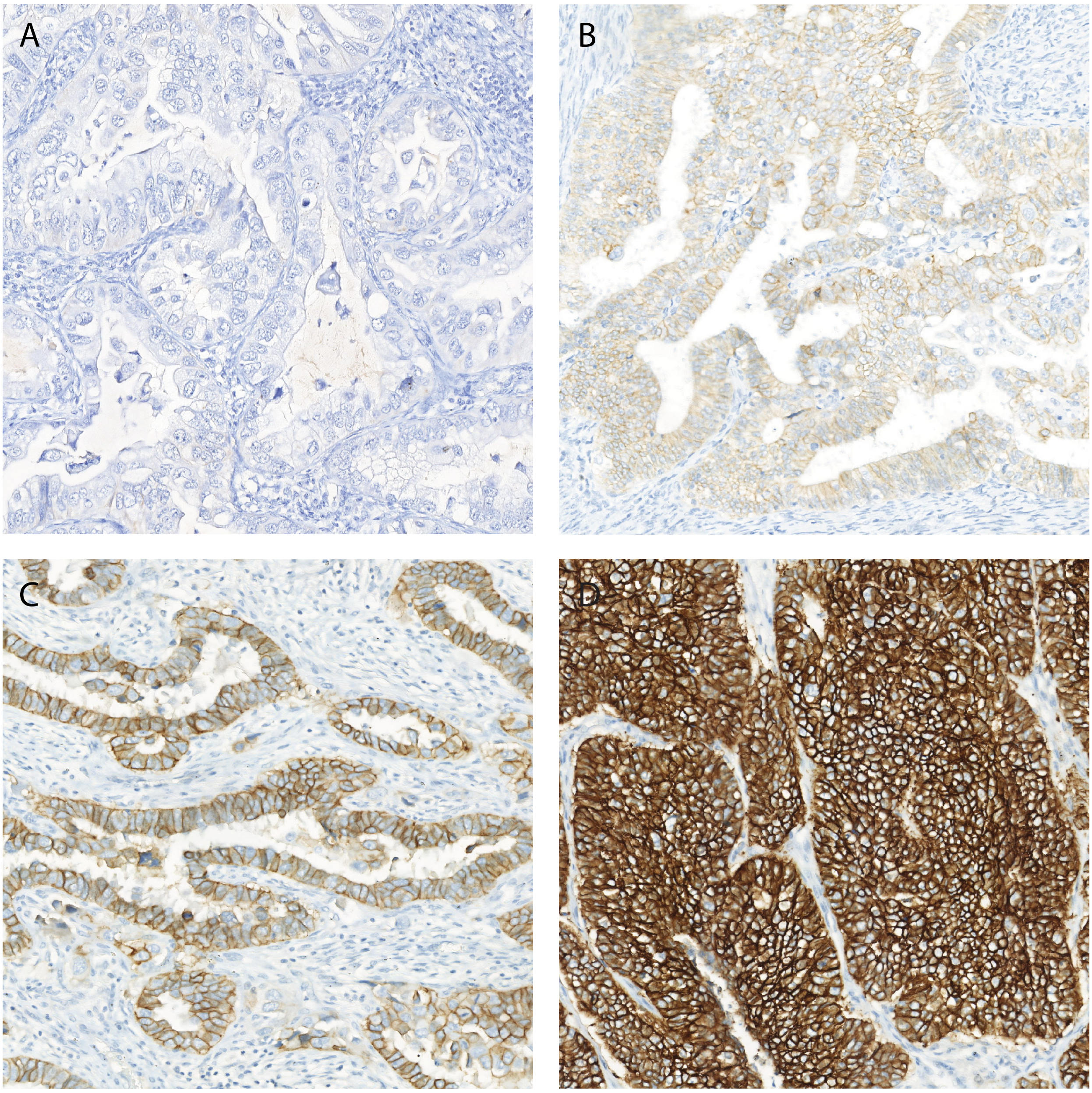

Supplement: Supplementary file 1 — Figure S1. Representative examples of absent, faint, moderate and strong membranous HER2 immunoreactivity in endometrial cancer. Representative examples of HER2 immunohistochemistry with (A) absent membranous immunoreactivity, (B) faint membranous immunoreactivity, (C) moderate membranous immunoreactivity, and (D) strong membranous immunoreactivity. [file HIS-79-533-s001.pdf]
