# Supplementary material for: Performance of a HER2 testing algorithm specific for p53‐abnormal endometrial cancer
Source: Histopathology. 2021 Jul 5;79(4):533–43. doi: 10.1111/his.14381 (PMC8518500; doi:10.1111/his.14381)

Figure S2. Two examples of potential pitfalls in HER2 IHC scoring in p53abn EC. (**A**) HER2 IHC of case #28 scored as IHC 1+ (by consensus), showing weak/moderate immunoreactivity in the basal membrane only (x40). (**B**) No amplification by DISH (HER2:CEP 17 ratio = 1.1; x120). (**C**) HER2 IHC of case #41 scored as IHC 0 (by consensus), showing strong nuclear and cytoplasmic staining (x40). (**D**) No amplification by DISH (HER2:CEP 17 ratio = 1.1; x90).

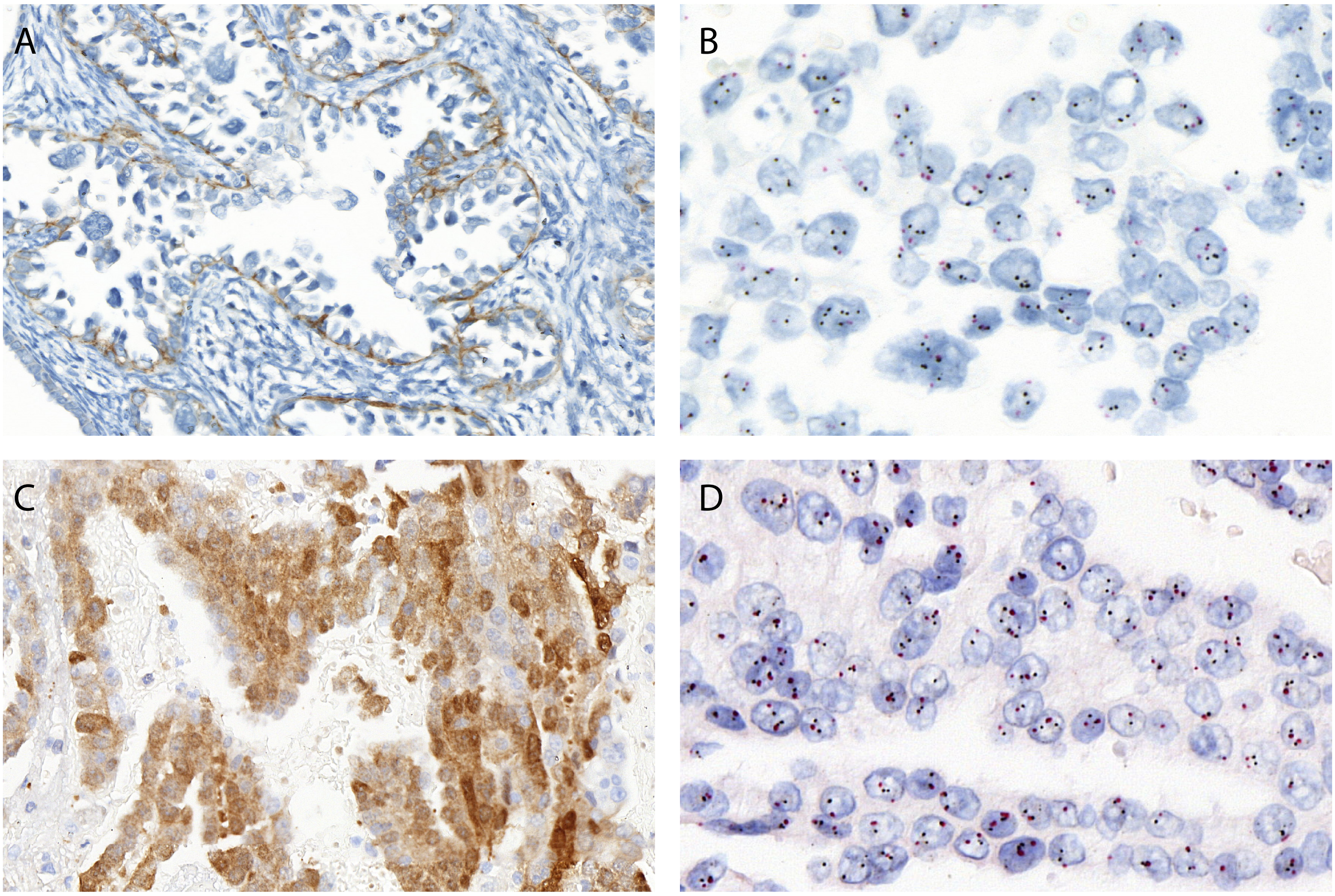

Supplement: Supplementary file 2 — Figure S2. Two examples of potential pitfalls in HER2 IHC scoring in p53abn EC. (A) HER2 IHC of case #28 scored as IHC 1+ (by consensus), showing weak/moderate immunoreactivity in the basal membrane only (×40). (B) No amplification by DISH (HER2:CEP 17 ratio = 1.1; ×120). (C) HER2 IHC of case #41 scored as IHC 0 (by consensus), showing strong nuclear and cytoplasmic staining (×40). (D) No amplification by DISH (HER2:CEP 17 ratio = 1.1; ×90). [file HIS-79-533-s003.pdf]
